# Multigene Mutation Profiling and Clinical Characteristics of Small-Cell Lung Cancer in Never-Smokers vs. Heavy Smokers (Geno1.3-CLICaP)

**DOI:** 10.3389/fonc.2019.00254

**Published:** 2019-04-17

**Authors:** Andrés F. Cardona, Leonardo Rojas, Zyanya Lucia Zatarain-Barrón, Alejandro Ruiz-Patiño, Luisa Ricaurte, Luis Corrales, Claudio Martín, Helano Freitas, Vladmir Cláudio Cordeiro de Lima, July Rodriguez, Jenny Avila, Melissa Bravo, Pilar Archila, Hernán Carranza, Carlos Vargas, Jorge Otero, Feliciano Barrón, Niki Karachaliou, Rafael Rosell, Oscar Arrieta

**Affiliations:** ^1^Clinical and Translational Oncology Group, Clinica del Country, Bogotá, Colombia; ^2^Foundation for Clinical and Applied Cancer Research, Bogotá, Colombia; ^3^Molecular Oncology and Biology Systems Research Group (Fox-G), Universidad El Bosque, Bogotá, Colombia; ^4^Clinical Oncology Department, Clínica Colsanitas, Bogotá, Colombia; ^5^Thoracic Oncology Unit, National Cancer Institute (INCan), Mexico City, Mexico; ^6^Department of Oncology, Hospital San Juan de Dios, San José, Costa Rica; ^7^Medical Oncology Group, Fleming Institute, Buenos Aires, Argentina; ^8^Department of Oncology, A.C. Camargo Cancer Center, São Paulo, Brazil; ^9^Instituto Oncológico Dr. Rosell (IOR), Quirón-Dexeus University Institute, Barcelona, Spain; ^10^Instituto Oncológico Dr. Rosell (IOR), Sagrat Cor Hospital, Barcelona, Spain; ^11^Cancer Biology and Precision Medicine Program, Catalan Institute of Oncology, Barcelona, Spain

**Keywords:** small-cell lung cancer, genome profile, next-generation sequencing, cancer in never-smokers, TP53, RB1, CYLD

## Abstract

**Objectives:** Lung cancer is a heterogeneous disease. Presentation and prognosis are known to vary according to several factors, such as genetic and demographic characteristics. Small-cell lung cancer incidence is increasing in never-smokers. However, the disease phenotype in this population is different compared with patients who have a smoking history.

**Material and Methods:** To further investigate the clinical and genetic characteristics of this patient subgroup, a cohort of small cell lung cancer patients was divided into smokers (*n* = 10) and never/ever-smokers (*n* = 10). A somatic mutation profile was obtained using a comprehensive NGS assay. Clinical outcomes were compared using the Kaplan-Meier method and Cox proportional models.

**Results:** Median age was 63 years (46–81), 40% were men, and 90% had extended disease. Smoker patients had significantly more cerebral metastases (*p* = 0.04) and were older (*p* = 0.03) compared to their non-smoker counterparts. For never/ever smokers, the main genetic mutations were *TP53* (80%), *RB1* (40%), *CYLD* (30%), and *EGFR* (30%). Smoker patients had more *RB1* (80%, *p* = 0.04), *CDKN2A* (30%, *p* = 0.05), and *CEBPA* (30%, *p* = 0.05) mutations. Response rates to first-line therapy with etoposide plus cisplatin/carboplatin were 50% in smokers and 90% in never/ever smokers (*p* = 0.141). Median overall survival was significantly longer in never smokers compared with smokers (29.1 months [23.5–34.6] vs. 17.3 months [4.8–29.7]; *p* = 0.0054). Never/ever smoking history (HR 0.543, 95% CI 0.41–0.80), limited-stage disease (HR 0.56, 95% CI 0.40–0.91) and response to first-line platinum-based chemotherapy (HR 0.63, 95% CI 0.60–0.92) were independently associated with good prognosis.

**Conclusion:** Our data supports that never/ever smoker patients with small-cell lung cancer have better prognosis compared to their smoker counterparts. Further, patients with never/ever smoking history who present with small-cell lung cancer have a different mutation profile compared with smokers, including a high frequency of *EGFR, MET*, and *SMAD4* mutations. Further studies are required to assess whether the differential mutation profile is a consequence of a diverse pathological mechanism for disease onset.

## Introduction

Lung cancer is the most common neoplasia worldwide. Aside from the high incidence, lung cancer also leads the list in terms of mortality, with the highest number of cancer-related deaths attributed to this tumor type. In this sense, lung cancer accounts for the lowest 5-year survival rate among other prevalent neoplasms, and therefore represents a significant healthcare burden worldwide (1, 2).

Nonetheless, lung cancer is a heterogeneous disease, and it can be categorized in terms of the major histological subtypes, which include adenocarcinoma, squamous cell, small cell and large cell carcinoma. Cigarette smoking is the best characterized lung cancer risk factor, and it is associated with a 19-fold increase in the risk of developing the disease, especially in women (3). Furthermore, it is responsible for 80–90% of lung cancer cases. Small cell lung carcinoma (SCLC) appears to have the strongest correlation with smoking status compared with other histological subtypes (4, 5). Previous studies indicate that 97.5% of patients with SCLC have a positive smoking history (6). Interestingly, the remaining 2.5% of SCLC cases represent non-smoker patients, and the pathogenic process remains unclear in this patient subgroup. Environmental tobacco smoke exposure, ionizing radiation, radon gas, inherited genetic susceptibility and oncogenic viruses are risk factors that have been implicated in the development of non-small cell lung carcinoma (NSCLC), unfortunately these associations have not been fully elucidated in SCLC (1, 7, 10).

Recent research has suggested that SCLC represents a distinct biological entity among non-smokers compared with smokers. In terms of clinical behavior, patients with extensive SCLC and a positive smoking history have shorter overall survival, despite the younger age compared to their non-smoker counterparts (6, 7). However, two reports from Korea and Spain have yielded contradictory results indicating a favorable association with survival outcomes for smoker patients and a detrimental effect in the never/ever smokers (8, 9). Molecular characteristics also tend to be different in the two patient subgroups. Due to the low frequency of SCLC in never smokers, the determination of the mutation status of the epidermal growth factor receptor (*EGFR*), routinely assessed in NSCLC patients, is a matter of debate. *EGFR* mutations are relatively rare in SCLC, with an estimated prevalence of approximately 7.1% (11). Several authors have shown that *EGFR* mutations are more prevalent among non-smokers. The determination of EGFR-mutation status is highly relevant since most mutations are sensitizing for treatment with tyrosine kinase inhibitors (TKIs), which have become the standard-of-care in *EGFR* mutated NSCLC (12, 13).

Despite the constant improvements in latter-generation TKIs, patients receiving this therapy eventually show progressive disease as a consequence of mechanisms of acquired resistance. Such mechanisms include the well-documented phenomenon of SCLC transformation from patients with a previous NSCLC tumor subtype (10, 14, 15). This transformation phenomenon occurs after approximately 19 months of TKI-treatment start, and causes a decrease in the overall survival even after treatment with cytotoxic chemotherapy (16, 17). Interestingly, previous studies have suggested that the clinical behavior of these transformed tumors could be different from either NSCLC and *de novo* SCLC (18). The underlying molecular biology relating to this phenomenon has not been fully established. Some authors suggest that a loss in the retinoblastoma protein (*Rb*) and mutations in *TP53* are the most important drivers. This hypothesis is further validated by Sun et al. who carried out a comprehensive next-generation sequencing (NSG) analysis in SCLC patients and found frequent mutations in *TP53, RB1*, and *PTEN*. These findings have strengthened the claim that *Rb* and *TP53* could be driver genes in SCLC regardless of smoking status. However, they also identified *FBXW7, RET*, and *VHL* mutations in never smoker patients, though these were not present in smoker patients (8). Further studies are needed to evaluate the role of these or other genetic alterations in the development and clinical behavior of SCLC among the non-smoker population. In the present cohort we followed a Hispanic population with SCLC; we evaluate and compare survival outcomes and molecular profiles among SCLC patients who presented with a heavy smoking history and those who were never/ever smokers.

## Patients and Methods

### Patients and Data Collection

**Figure 1:**
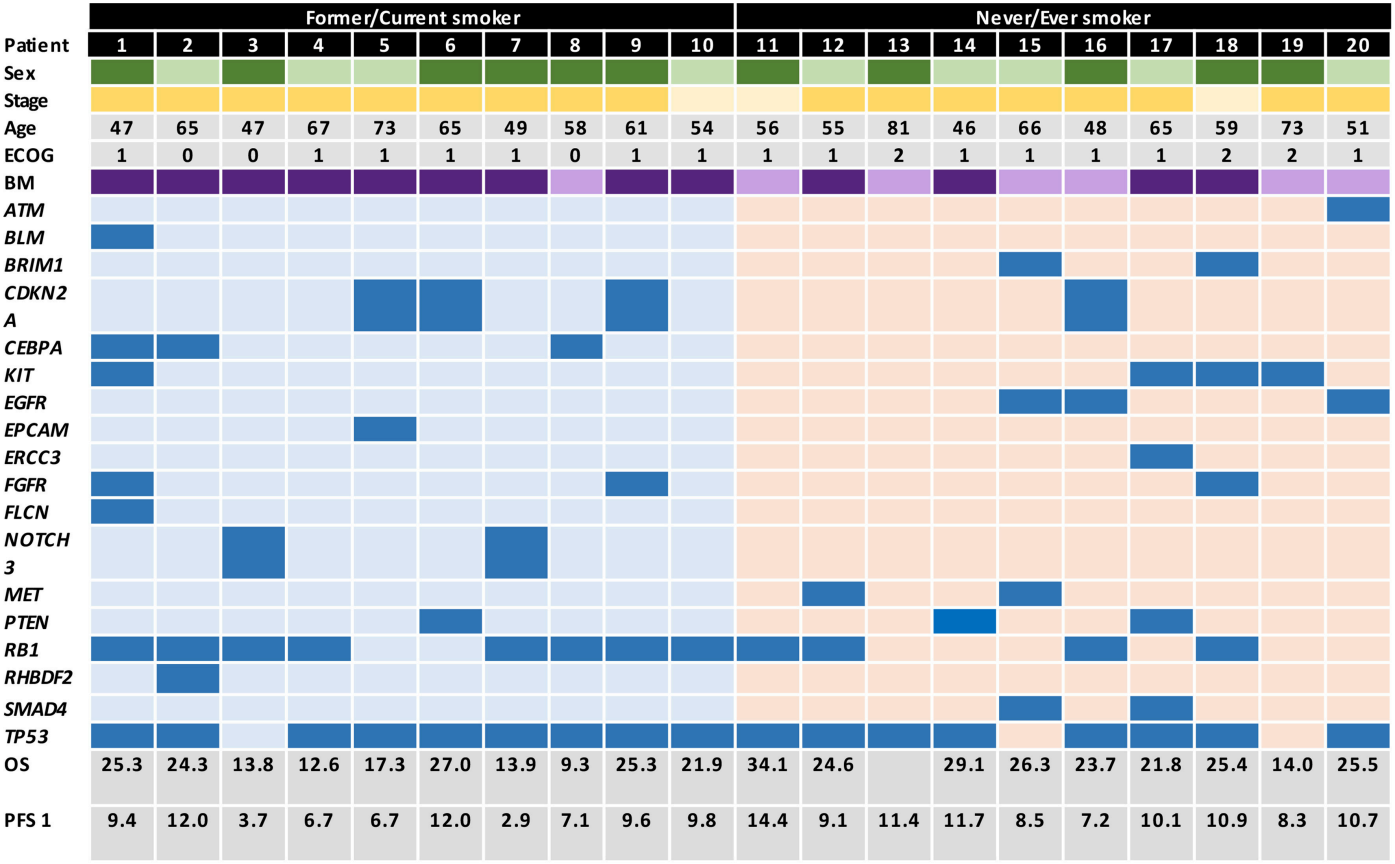
Graphical distribution of the genomic profile in heavy smokers and never/every smokers SCLC patients.

All patients (*n* = 88) who were histologically diagnosed with advanced or metastatic (American Joint Committee on Cancer, AJCC homologated to stages IIIB or IV) SCLC and treated at two institutions in Bogotá, Colombia between January 2010 and June 2016 were assessed for eligibility. Among these, 20 patients (10 smokers and 10 non-smokers) met inclusion criteria, had a complete follow-up and were matched as close as possible by clinical and pathological variables (Supplementary Figure 1 includes the study outline). An Institutional Review Board and Privacy Board waiver was obtained to facilitate retrospective collection of clinical-pathologic and molecular data (Geno1.3-CLICaP Platform – RN18034-16, Clínica del Country, Bogotá, Colombia). Clinical data collected were: age, gender, tobacco exposure (ever smoker was defined as a subject who self-reported as never exposed to tobacco and never smoker was defined as those who report having smoked ≤100 cigarettes in their lifetime), ECOG performance status, TNM (19), number and sites of metastases, and presence of brain involvement. The diagnosis of SCLC was confirmed through histologic and immunohistochemical (IHC) testing performed by two expert pathologists (all slides were double checked by both observers) for all included patients. None of the cases had a mixed histology component (including adenocarcinoma or atypical carcinoid). Pathologic data included the expression of TTF1, chromogranin, synaptophysin, CD56, Ki67, and PDL1 (22C3) (Supplementary Figure 2 includes representative images of the immunohistochemistry of a tumor from a patient with tobacco exposure and a tumor from a patient without exposure to tobacco). In addition, we included the mutation status of *EGFR*, the estimation of tumor mutational burden (TMB) and the presence of other mutations explored by NGS (TruSight Tumor™ 170) (Figure 1 includes the database with genomic raw data and clinical variables).

### Next-Generation Sequencing and EGFR Mutation Test

The archived tissue samples from heavy smoker and never/ever smoker patients were histologically reviewed and analyzed with NGS. DNA and RNA were extracted from formalin-fixed paraffin-embedded (FFPE) samples using the Qiagen AllPrep kit. Following extraction, DNA samples were quantified using Qubit and RNA samples were quantified using BioAnalyzer. Quality evaluation was performed thereafter. DNA samples were assessed by qPCR using the Illumina FFPE QC Kit (WG-321-1001) along with a control cell line sample with a known input mass. Following quantification and quality assessment, samples that met the minimum input threshold (3.3 ng/μl for DNA, 4.7 ng/μl for RNA), regardless of quality, were processed through the TruSight Tumor™ 170 assay. Briefly, DNA samples were sheared for library preparation and RNA samples were converted to cDNA. Subsequently, both sample types were run in parallel through library preparation followed by a hybrid capture enrichment targeting 170 key cancer genes. Samples were evaluated for performance based on quality control (QC) metrics established during the development of the TruSight Tumor™ 170 assay. For never/ever-smoker samples an independent *EGFR* mutation analysis was performed to confirm the results of NGS using the Cobas® v2 probe. Briefly, tumor specimens and genomic DNA was isolated using the Cobas® DNA Sample Preparation Kit. A manual specimen preparation based on nucleic acid binding to glass fibers was performed. The deparaffinized 5-μm section of an FFPET specimen was lysed by incubation at an elevated temperature with a protease and chaotropic lysis/binding buffer (it releases nucleic acids and protects them from DNases). Subsequently, isopropanol was added to the lysis mixture and was centrifuged in a column with a glass fiber filter insert. During centrifugation, the genomic DNA was bound to the surface of the glass fiber filter. Unbound substances, such as salts, proteins and other cellular impurities, were removed by centrifugation. The adsorbed nucleic acids were washed and then eluted with an aqueous solution. The amount of genomic DNA was spectrophotometrically determined and adjusted to a fixed concentration added to the amplification/detection mixture. The target DNA was amplified and detected on the Cobas® Z480 analyzer using the amplification and detection reagents provided in the Cobas® EGFR Test.

### Immunohistochemistry (IHC) for PDL1

IHC analysis was carried out in tissue sections that were previously deparaffinied (EZprepTMx10) in an oven for 30 min at 60°C followed by three serial xylene incubations. Sections were then rehydrated in graded alcohols and subjected to antigen retrieval using XS Tris Buffered Saline with Tween 20 and boiled for 20 min. Rabbit monoclonal primary PD-L1 antibody (22C3) was added and further processed using 4 mm-thick FFPE tissue sections on a Benchmark XT autostainer (Ventana Medical System) with standard antigen retrieval methods. The Signal Stain DAB substrate kit (#8959) was used according to the manufacturer's instructions. Human placenta was included as positive control for endogenous PD-L1. All IHC stained sections were evaluated and scored by two PA and discrepancies in interpretation of scoring were resolved by consensus. Tumors with ≥1% of tumor cells stained (membrane or cytoplasm staining) were considered positive for PD-L1. The expression of PD-L1 was evaluated according to the intensity of the staining and scored using the following system: 0, negative; 1, weak expression; 1–49%, moderate expression; and 50–100%, strong expression.

### Statistical Analysis

For descriptive purposes, continuous variables were summarized as arithmetic means, medians and standard deviations. Categorical variables were reported as proportions with 95% confidence intervals (95% CIs). Inferential comparisons were performed using Student's *t*-test. χ^2^ or Fisher's exact test were used to assess the significance among categorical variables. The time-to-event variables obtained from the Kaplan-Meier method were determined by log-rank tests. Statistical significance was considered as *p* ≤ 0.05 using a two-sided test. All of the statistical analyses were performed using SPSS version 23.0 (SSPSS, Inc., Chicago, IL, US).

## Results

### Patient Characteristics

A total of 88 patients presented with SCLC between January 2010 and June 2016. Among these, 12.5% were never/ever smokers, accounting for an estimated incidence of 1.6% cases/year. From these, 20 patients (10 smokers and 10 non-smokers) met inclusion criteria, had a complete follow-up and were matched as close as possible by clinical and pathological variables, and were included in this study. The characteristics of the sequenced cohort are represented in Table 1. Median age for the entire population was 63 years (range 46–81), 40% of the patients were male and 90% had extended disease. The mean tobacco consumption in the smoker group was estimated at 41.3 (SD ± 20) packs/year. Additionally, 70% of them smoked 30 packs/year or more before diagnosis. In terms of clinical differences between smokers and ever/never smokers, a statistically significant difference in age of presentation was observed, favoring a younger age for SCLC smoker patient group [mean difference of 7.5 years (57.5 vs. 65 years); *p* = 0.02]. As well as a female gender, which was significantly more predominant in the never/ever smoker population (80 vs. 40%; *p* = 0.003). In terms of disease burden, a higher rate of central nervous system metastatic involvement was noted in the heavy smoker patients (90 vs. 40%; *p* = 0.04).

**Table 1:** Clinical and molecular characteristics.

**Variable**	**All cases**	**Never/ever smokers**	**Heavy smokers**	***P*-value^∫^**
	***N* = 20 (%)**	***N* = 10 (%)**	***N* = 10 (%)**	
**Age (mean)**	63.0 (r, 46–81)	57.5 (r, 46–81)	65.0 (r, 54–77)	0.02
<65 years	14 (70.0)	6 (60.0)	8 (80.0)	
>65 years	6 (30.0)	4 (40.0)	2 (20.0)	
**Gender**				
Female	12 (60.0)	8 (80.0)	4 (40.0)	0.003
Male	8 (40.0)	2 (20.0)	6 (60.0)	
**ECOG performance status**				
0	3 (15.0)		3 (30.0)	0.44
1	14 (70.0)	7 (70.0)	7 (70.0)	
2	3 (15.0)	3 (30.0)	–	
**Disease extension**				
Extended disease	18 (90.0)	9 (90.0)	9 (90.0)	0.51
Limited disease	2 (10.0)	1 (10.0)	1 (10.0)	
**Tumor size**				
Mean ± SD (mm)	45.9 ± 13.0	42.3 ± 12.5	49.5 ± 13.1	0.08
**T stage**				
1	4 (20.0)	3 (30.0)	1 (10.0)	0.56
2	9 (45.0)	5 (50.0)	4 (40.0)	
3	6 (30.0)	2 (20.0)	4 (40.0)	
4	1 (5.0)	–	1 (10.0)	
**N stage**				
0	3 (15.0)	1 (10.0)	2 (20.0)	0.4
1	8 (40.0)	4 (40.0)	4 (40.0)	
2	6 (30.0)	3 (30.0)	3 (30.0)	
3	3 (15.0)	2 (20.0)	1 (10.0)	
**M stage**				
0	2 (10.0)	1 (10.0)	1 (10.0)	0.70
1	18 (90.0)	9 (90.0)	9 (90.0)	
**Tobacco exposure**				
Packs/year	–	–	41.3 ± 20.0	–
30 pack-years or more before diagnosis	–	–	7 (70.0)	
Current smoker	–	–	10 (100.0)	
**Site of metastases**				
2	13 (65.0)	6 (60.0)	7 (70.0)	0.65
3	5 (25.0)	3 (30.0)	2 (20.0)	
≥4	2 (10.0)	1 (10.0)	1 (10.0)	
**Main site of metastasis**				
Pleural/Lung	13 (65.0)	7 (70.0)	6 (60.0)	0.53
Bone	3 (15.0)	1 (10.0)	2 (20.0)	
Suprarenal	1 (5.0)	–	1 (10.0)	
Nodal	1 (5.0)	1 (10.0)		
Brain metastases^*^				
Present	13 (65.0)	4 (40.0)	9 (90.0)	0.04
Absent	7 (35)	6 (60.0)	1 (10.0)	
**Pathological sample site**				
Primary	19 (95.0)	9 (90.0)	10 (100.0)	0.82
Metastases	1 (5.0)	1 (10.0)		
**TTF1**				
Positive	17 (85.0)	9 (90.0)	8 (80.0)	0.59
Negative	3 (15.0)	1 (10.0)	2 (20.0)	
**CD56**				
Positive	19 (95.0)	10 (100.0)	9 (90.0)	0.62
Negative	1 (5.0)	–	1 (10.0)	
**Chromogranin**				
Positive	19 (95.0)	9 (90.0)	10 (100.0)	0.78
Negative	1 (5.0)	1 (10.0)		
**PDL1**				
<1%	10 (50.0)	6 (60.0)	4 (40.0)	0.06
1–49%	7 (35.0)	3 (30.0)	4 (40.0)	
>50%	3 (15.0)	1 (50.0)	2 (20.0)	
**KI67**				
70	2 (10.0)	1 (10.0)	1 (10.0)	0.83
80	6 (30.0)	3 (30.0)	3 (30.0)	
90	11 (55.0)	5 (50.0)	6 (60.0)	
100	1 (5.0)	1 (10.0)		
**EGFR common sensitizing mutation**				
Present	3 (15.0)	3 (30.0)		0.01
Absent	17 (85.0)	7 (70.0)	10 (100.0)	
**EGFR sensitizing mutation**				
L858R	1 (5.0)	1 (10.0)		–
T446K	1 (5.0)	1 (10.0)		
Del19	1 (5.0)	1 (10.0)		
**TMB**				
<7	5 (25.0)	1 (10.0)	4 (40.0)	0.18
8–14	10 (50.0)	5 (50.0)	5 (50.0)	
>15	5 (25.0)	4 (40.0)	1 (10.0)	

∫, * *p = 0.05*.

### Treatment

All patients were treated with a first line regimen of platinum/etoposide combination chemotherapy. Overall response rate (ORR) was 70% among the entire population, however this was higher for the never/ever smoker group (90%) compared with the smoker patient group (50%), however this difference was not statistically significant (*p* = 0.141). Second line treatment was administered to all patients after eventual disease progression. Platinum/etoposide combination re-challenge was given to 8 patients (3 smokers and 5 never/ever smokers), platinum/irinotecan was used in 7 patients (4 smokers and 3 never/ever smokers), irinotecan monotherapy was given to 2 patients (1 smoker and 1 never/ever smoker), topotecan monotherapy was used in 2 (2 smokers) and erlotinib was given to 1 never/ever smoker patient. No statistically significant differences were observed when comparing ORR in this setting (*p* = 0.179). No complete responses were observed, although a tendency toward more partial responses was documented in the never/ever smokers group (70 vs. 30%; *p* = 0.054). A final third line was offered to 19 patients, all but one patient in the heavy smokers group. ORR for the never/ever smoker group was 60% against 11% in the heavy smokers group (*p* = 0.171). Topotecan was offered to 5 patients (2 smokers and 3 never/ever smokers), a taxane to 8 (5 smokers and 3 never/ever smokers), irinotecan to 4 (1 smoker and 3 never/ever smokers), amrubicin to 1 heavy smoker patient and cisplatin/irinotecan to 1 never/ever smoker case.

### Molecular Characteristics

Molecular characteristics of all patients are summarized in Table 1 and Figure 1. There were no differences in terms of expression of TTF1, CD56, or chromogranin. Additionally, patients had a similar profile in terms of PD-L1 expression, in never/ever smokers 60, 30, and 10% had PD-L1 expression of <1, 1–49, and >50%, respectively. In the case of smoker patients, 40, 40, and 20% had PD-L1 expression of <1, 1–49, and >50%, respectively. EGFR mutations were significantly higher in the never/ever smoker patient group compared with the smoker group (30 vs. 0%; *p* = 0.01). However, TMB was similar across both patient groups, with 50% of patients in both groups presenting with a TMB ranging from 8 to 14, while 40% of patients in the never/ever smoker had a TMB >15, compared to only 10% in the smoker group. However, these differences were not statistically significant.

Among never/ever smokers, the most frequent genetic mutations were *TP53* (80%), *RB1* (40%), *CYLD* (30%), *EGFR* (30%), *MET* (20%), *SMAD4* (20%), and *BRIP1* (20%). However, none of the smoker patients had mutations in *EGFR, MET* or *SMAD4*, but presented a greater involvement of *RB1* (80%, *p* = 0.04), *CDKN2A* (30%, *p* = 0.05), *CEBPA* (30%, *p* = 0.05), *FANCG* (20%), *GATA2* (20%), and *PTEN* (20%) mutations.

### Survival Outcomes

**Figure 2:**
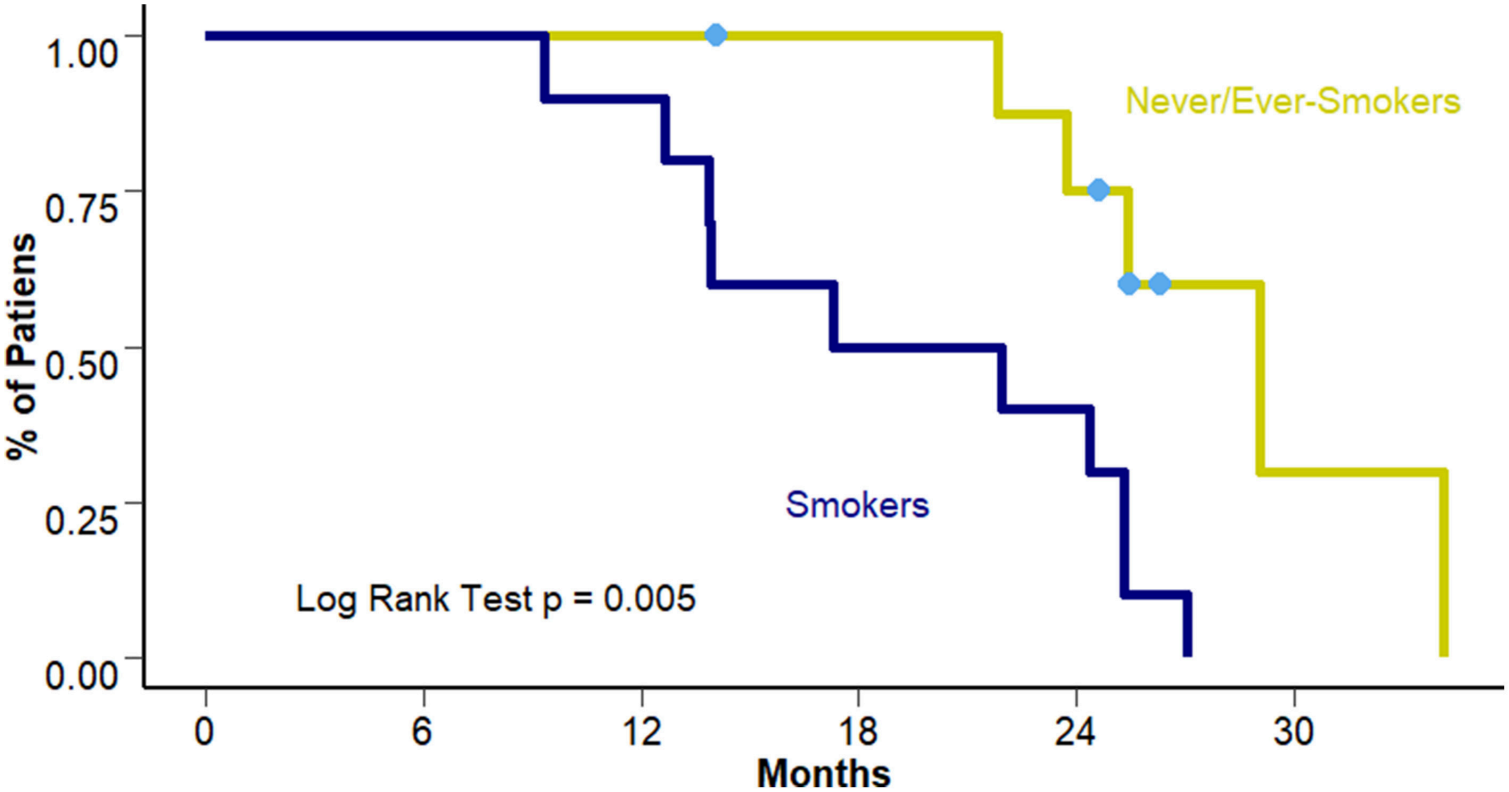
OS according to smoking status.

Overall Survival for the whole cohort reached a median of 25.3 months (95% CI 21.8–27.4 months). When comparing differences between smokers and never/ever smokers a benefit of approximately 10 months was observed in the never/ever smokers group (19.6 vs. 29.1 months [95% CI 4.8–29.7 and 23.5–34.6 months] respectively; *p* = 0.005) (Figure 2).

**Figure 3:**
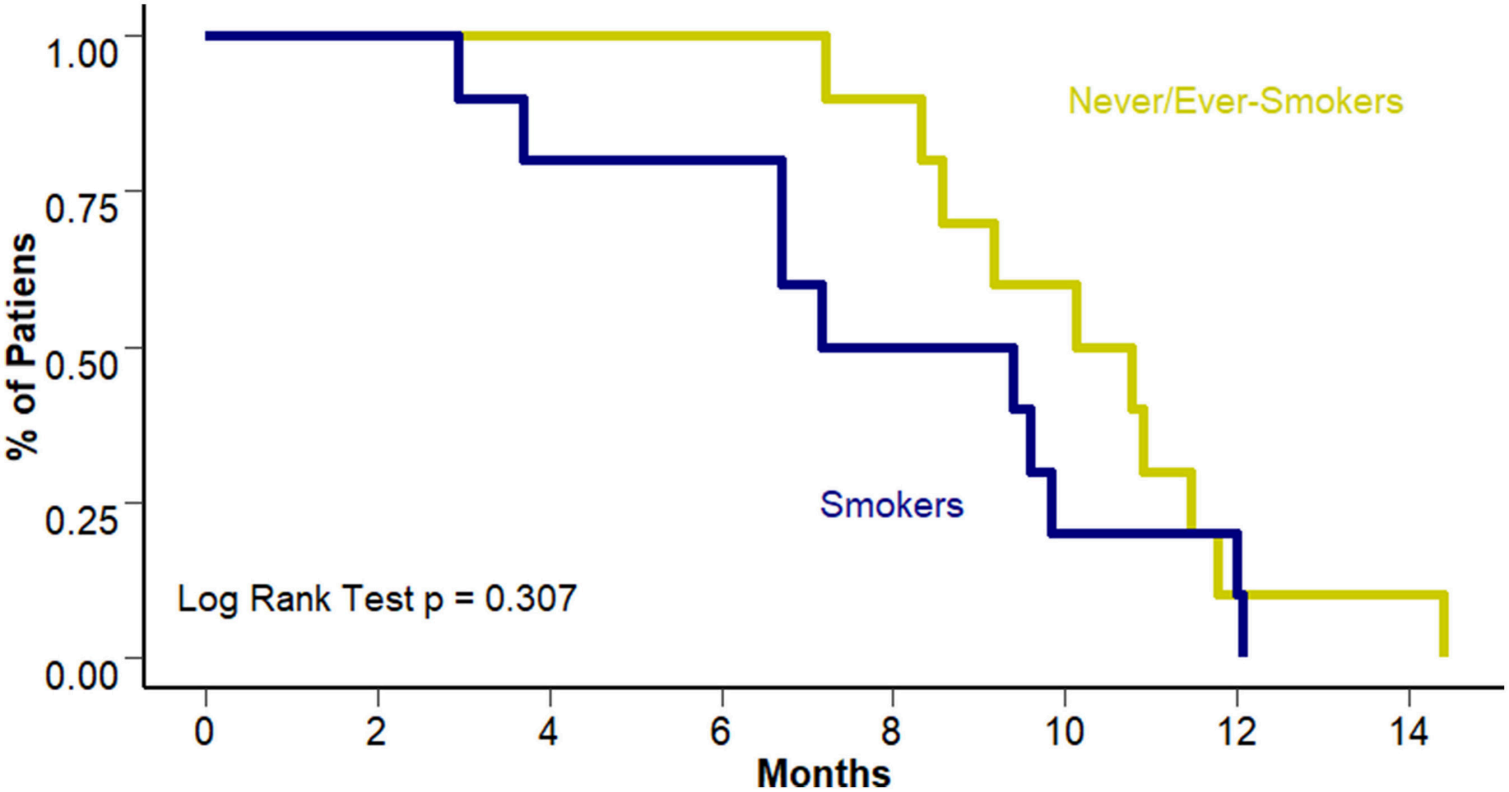
PFS to first line according to smoking status.

**Figure 4:**
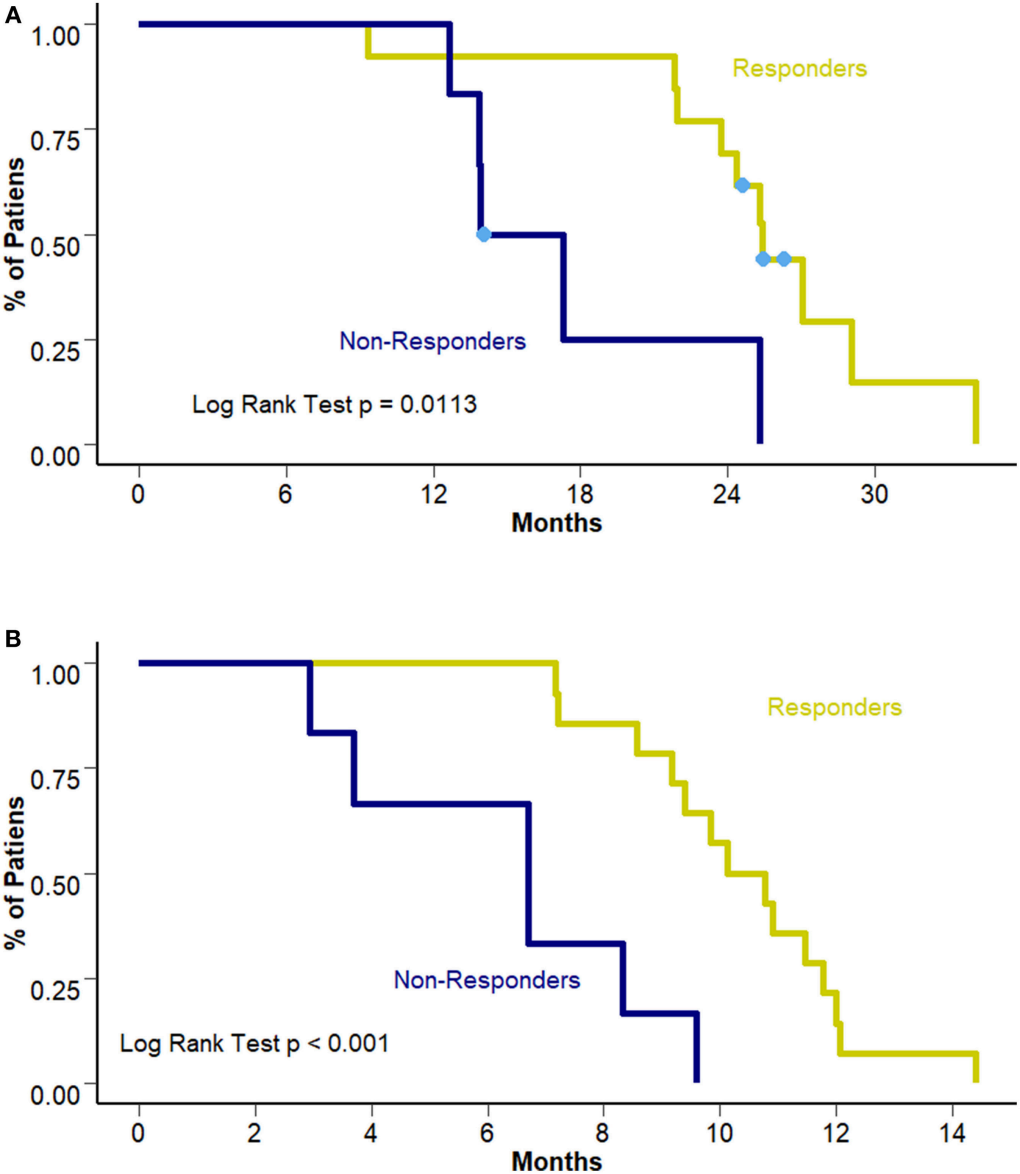
**(A)** OS according to response to first line treatment. **(B)** PFS according to response to first line treatment.

Interestingly, this benefit was not observed in terms of PFS to first line treatment (8.28 vs. 10.45 months [95% CI 6.7–10.3 and 8.57–12.3 months]; *p* = 0.307). Figure 3 presents PFS to first line treatment according to smoking status. Response to first line treatment was also associated with a benefit in OS (25.4 vs. 15.6 months [95% CI 23.8–28.2 and 13.9–18.4 months, respectively]; *p* = 0.0113) and PFS (6.7 vs. 10.4 months [95% CI 9.4–12.1 and 3.7–9.1 months, respectively]; *p* < 0.001) among never/ever smokers. Survival curves according to overall response are presented in Figures 4A,B. The multivariate analysis showed that never/ever-smoking history (HR 0.543, 95% CI 0.41–0.80) and limited stage disease (HR 0.56, 95% CI 0.40–0.91) were independently associated with improved survival.

## Discussion

There are clear clinical and biological differences in patients with SCLC who have a history of tobacco exposure and those who are never/ever smokers. First, there is a demographic disparity. Our data shows that never/ever smoker patients tended to be younger and female compared with their heavy smoker counterparts. Other recently published cohorts have shown similar results in regard to gender predispositions. In a study conducted by Torres-Duran et al. 19 patients with SCLC who were never smokers were followed. Almost all were female patients (*n* = 18), with a median age of 75 years (9). Similarly, another study conducted at the Memorial Sloan Kettering Cancer Center (MSKCC) which included 19 never smoker patients with *de novo* SCLC, reported a predisposition in the female population under 65 years (20). Furthermore, a sampled cohort of 8 Japanese patients with similar characteristics as the aforementioned cohorts, revealed a higher incidence in elder patients (21). Compared to our results, never smoker patient characteristics differ in age at presentation, suggesting a disparity in risk factor exposure or tumor biological background between the aforementioned cohorts.

The patients in the present study have a much longer survival compared with previous reports. The analysis of the Californian Cancer Registry, comprising 4,782 patients with SCLC with and without smoking history sampled between 1991 and 2005, calculated a median overall survival of 5 months, though it accounted for different ethnic backgrounds and diverse treatment strategies (22). However, it is important to highlight that comparing our results to the previously reported data is difficult, since important differences exist in terms of sample size, treatment strategies and ethnicity. For instance, the therapeutic schemes and strategies of the California patients are not well-described. Second, our cohort of patients was heavily treated, with most patients receiving at least three lines of therapy. Additionally, treatment strategies were multimodal with many therapeutic combinations. To further complicate comparisons, the management of disease recurrences is complex, with a dismal survival even after aggressive treatment. Current guidelines recommend re-challenge treatments with the initial therapeutic regimen as a second line chemotherapy, when progression presents after 6 months of treatment start (23). Single agent chemotherapy seems to be effective to prevent early progression. Proposed agents include topotecan, paclitaxel and amrubicin (24–26). Combination chemotherapy is a good alternative, platinum/etoposide or irinotecan has proven to prolong OS. Patients that undergo this regimen after two lines of treatment reached a median OS of 18.2 (27). Present results suggest that multiple lines of chemotherapy regimens are responsible for the outcomes.

According to our results and to the previously cited publications, smoking seems to be associated with worse outcomes, specifically shorter OS. Remarkably, data from our cohort shows that patients who presented with a smoking history had a higher incidence of brain metastases. Hence, brain metastases likely represent a detrimental factor for survival in this subset of patients.

The molecular landscape in SCLC patients seems to differ according to their smoking status. The prevalence of *EGFR* mutations on never/ever smokers was estimated to be approximately 7% and it correlated with a better survival. Overall *EGFR* mutation prevalence in SCLC is around 2% (11). Additionally, the frequency of classical types of *EGFR* mutations (Del 19, L858R) appear to be lower than in NSCLC (~7.5% of EGFR mutated each), favoring rare mutation types, including Exon 18 (G719D/S, G696R, S695N/D, N700D, I715F, L688F, P694L), Exon 19 (K757N, A755V, V742I, E736K, N756Y, E749K, P753L, A755T), Exon 20 (T790M, H773R, S768R/N, R776H/C, G796D, D807N, R803W/Q, Y813C, G810S, A763T, G779D, Q791R, C781Y, N771S) and Exon 21 (L858V, G874R, K867E) (11). Recent large-scale sequencing studies including 175 whole genomes, 95 transcriptomes and 142 SNP arrays of human SCLCs revealed a mere total of four *EGFR* mutations, T446K, I643V, H893R, and L1167V, all of uncertain clinical relevance (28–30).

Interestingly, SCLC exhibits extremely high mutation rates at around 8.62 mutations per million base pairs, with 28% of tumors exhibiting C:G>A:T transversions. As previously acknowledged, *RB1* and *TP53* are key elements in the molecular biology of SCLC (27). Inactivating mutations in these genes affect up to 65 and 90% of samples, respectively. In the case of *RB1* mutations, an alteration in the exon-intron junction is the most prevalent phenomenon. This in turn causes protein damage splice events. TP53, on the other hand, is affected by a missense mutation in the coding sequence for the functional DNA binding domain. Additionally, *TP53* and *RB1* were lost in both alleles in 100 and 93% of the cases, respectively (28).

Heavy smokers and never/ever smokers have a high prevalence of *TP53* mutations. In contrast, *RB1, CDKN2A*, and *CEBPA* are more prevalent among smokers. The data we present in terms of mutation prevalence resembles previously published cohorts. Unfortunately, the vast majority of patients analyzed in previous studies were smokers (31). *RB1* is the most relevant mutational difference between smoker and non-smoker SCLC patients. Consequently, smoker patients with wild type *RB1* should behave similarly to non-smoker patients. *RB1*, responsible for the regulation of the cell cycle, is also involved in response to DNA damaging agents. Thus, a disruption in this molecule would sensitize the tumor cell to chemotherapy and offer a longer survival outcome (32). These findings were validated in an analysis of 39 patients with ED-SCLC in which 42% of samples had mutated *RB1*. OS for wild-type patients was shorter at 9.1 vs. 11.7 months when the mutation was present (*p* = 0.04) (33). Nevertheless, the present study shows a benefit in survival for never/ever smoker patients regardless of *RB1* status. This could represent a differential disease biology between never/ever smokers and smoker patients with SCLC, which might be responsible for the differences in outcomes, independent to the *RB1* mutational status. However, this observation requires validation from more studies in order to fully elucidate this relationship.

TMB, a quantification of mutations in the tumor genome has been strongly associated with the response to immunotherapy with checkpoint inhibitors such as pembrolizumab or ipilimumab/nivolumab (34, 35). A pooled analysis of the cohorts of the CheckMate-032 study evaluated treatment with nivolumab or combination of nivolumab/ipilimumab. Researchers performed a TMB evaluation in 211 patients from the two arms of the study. Additionally, and as a complementary evaluation, PD-L1 expression was quantified. Results yielded an ORR with combination therapy of 46.2% for high TMB. This observation correlated with OS: patients with high TMB achieved a median of 22 months, compared to 3.6 and 3.4 months in the medium and low TMB. On the other hand, PD-L1 expression was not found to significantly correlate with major clinical endpoints. Although in our cohort never/ever smoker and smoker patients had similar TMB, the majority of patients with high TMB were elderly smokers.

Limitations of the study included its retrospective nature. Further, it included a high incidence of never/ever smokers SCLC patients compared to previous reports. This could potentially cause a selection bias. Additionally, this phenomenon opens the possibility of a different disease biology or currently unknown risk factor present in the Colombian population, compared with previously studied populations from other ethnic backgrounds. A larger, multicentric and multiethnic study should be designed in order to prospectively validate these results.

## Conclusions

Never/ever smokers with SCLC have a better prognosis compared with their smoker counterparts. *EGFR, MET*, and *SMAD4* are frequent mutations among SCLCs of never/ever smokers, and *RB1, CDKN2A*, and *CEBPA* among heavy smokers.

## Ethics Statement

An Institutional Review Board and Privacy Board (Clinica del Country) waiver was obtained to facilitate retrospective collection of clinical-pathologic and molecular data. No interventional therapeutics or invasive tests were performed outside normal clinical practice. Samples were collected from biopsy specimens extracted outside of a clinical study.

## Author Contributions

Study design and data collection: AC, LRo, ZZ, HC, CV, JO, FB, NK, RR, and OA. Molecular, pathological and clinical analysis: JR, JA, MB, PA. Statistical Analysis, and manuscript drafting: AC, LRi, AR, LC, CM, HF, VC. All authors approved the final and submitted version of the manuscript.

### Conflict of Interest Statement

The authors declare that the research was conducted in the absence of any commercial or financial relationships that could be construed as a potential conflict of interest.

## Supplementary Material

Supplementary Figure 1 Study schema.

Supplementary Figure 2 Representative images of the immunohistochemistry of a tumor from a patient with tobacco exposure and a tumor from a patient without exposure to tobacco.

## Abbreviations

SCLC: Small-cell lung cancer
NSCLC: Non-small cell lung cancer
EGFR: Epidermal Growth Factor Receptor
TKI: Tyrosine kinase inhibitor.
