# Supplementary figures and images for: Multigene Mutation Profiling and Clinical Characteristics of Small-Cell Lung Cancer in Never-Smokers vs. Heavy Smokers (Geno1.3-CLICaP)
Source: Front Oncol. 2019 Apr 17;9:254. doi: 10.3389/fonc.2019.00254 (PMC6481272; doi:10.3389/fonc.2019.00254)

## Supplementary figure 1 | Study schema

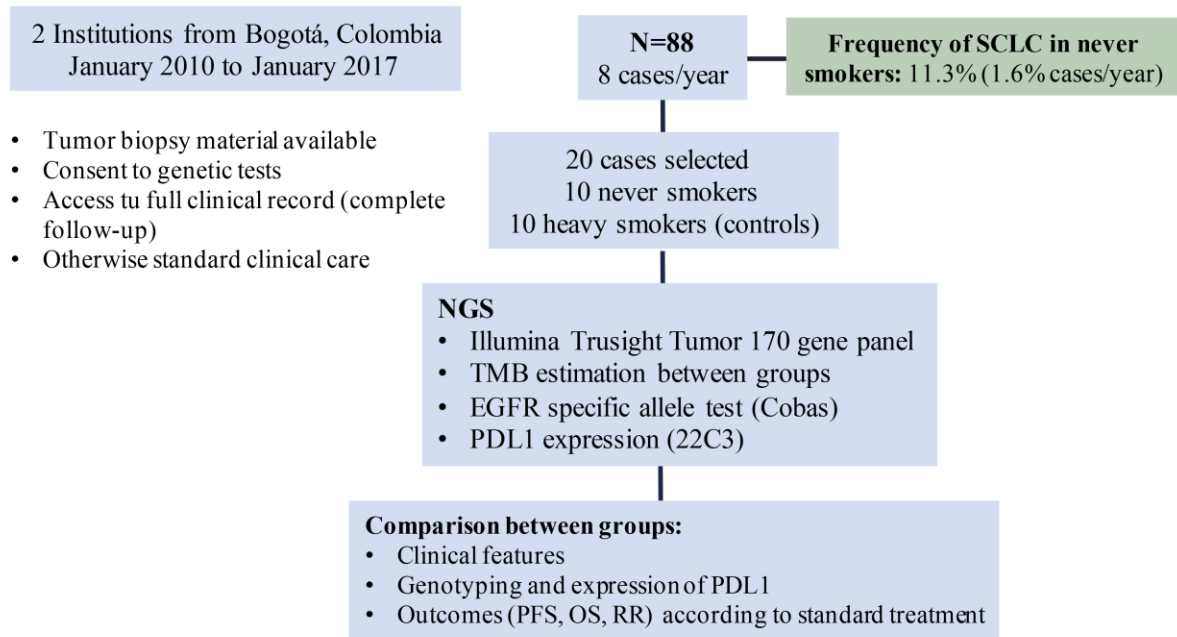

Supplement: Supplementary Figure 1 — Study schema. [file Image_1.pdf]
