# Supplementary material for: Multigene Mutation Profiling and Clinical Characteristics of Small-Cell Lung Cancer in Never-Smokers vs. Heavy Smokers (Geno1.3-CLICaP)
Source: Front Oncol. 2019 Apr 17;9:254. doi: 10.3389/fonc.2019.00254 (PMC6481272; doi:10.3389/fonc.2019.00254)

Supplementary figure 2 | Representative images of the immunohistochemistry of a patient with and without exposure to tobacco. Example of biopsy specimen with uniform diagnosis of SCLC in a current (A) and ever smoker patients (E) (hematoxylin and eosin stain; original magnification, ×100). (B and F) TTF1 immunohistochemistry (IHC) staining with abundant membranous pattern (original magnification, ×100). (C and G) CD56/NCAM IHC staining with cytoplasmic pattern (original magnification, ×100). (D and H) Chromogranin IHC staining (original magnification, ×100).

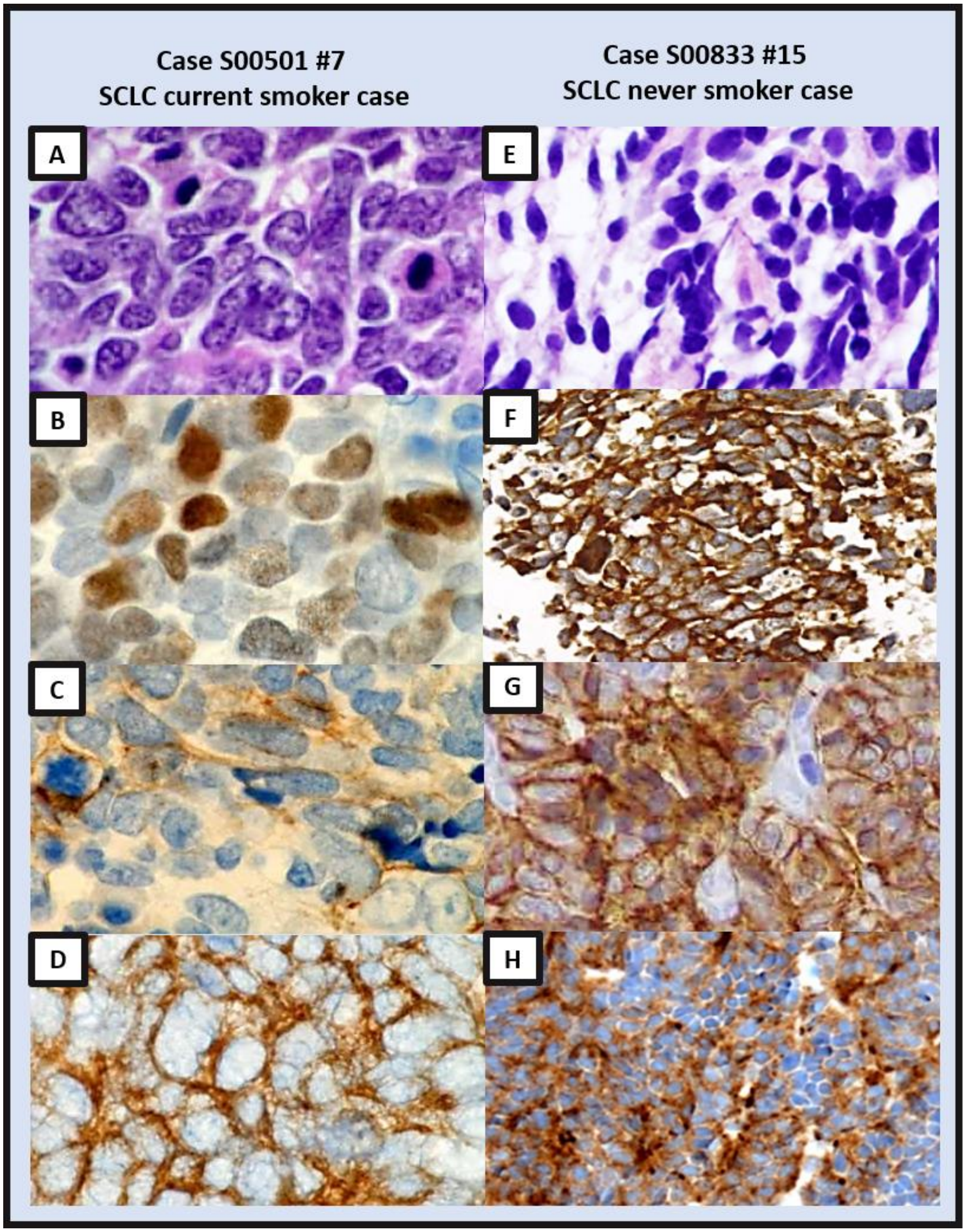

Supplement: Supplementary Figure 2 — Representative images of the immunohistochemistry of a tumor from a patient with tobacco exposure and a tumor from a patient without exposure to tobacco. [file Image_2.pdf]
